# Production of an anti-Aβ antibody fragment in *Pichia pastoris* and *in vitro* and *in vivo* validation of its therapeutic effect

**DOI:** 10.1371/journal.pone.0181480

**Published:** 2017-08-03

**Authors:** Laia Montoliu-Gaya, Gisela Esquerda-Canals, Silvia Bronsoms, Sandra Villegas

**Affiliations:** 1 Protein Folding and Stability Group, Departament de Bioquímica i Biologia Molecular, Universitat Autònoma de Barcelona, Bellaterra, Barcelona, Spain; 2 Departament de Biologia Cel·lular, de Fisiologia i d’Immunologia, Unitat de Citologia i d’Histologia, Universitat Autònoma de Barcelona, Bellaterra, Barcelona, Spain; 3 Servei de Proteòmica i Biologia Estructural, Universitat Autònoma de Barcelona, Bellaterra, Barcelona, Spain; Weizmann Institute of Science, ISRAEL

## Abstract

ScFv-h3D6 has been shown as an efficient therapy in the 3xTg-AD mouse model of Alzheimer’s Disease. Because one of the major bottlenecks for the therapeutic uses of proteins produced in *Escherichia coli* is their potential contamination with endotoxins, LPS were extensively removed by a rather low-efficient, expensive, and time-consuming purification step. In addition, disulfide scrambling is favored in the reducing bacterial cytoplasm albeit the use of reductase deficient strains. To overcome these hurdles, as well as to improve the yield, the yeast *Pichia pastoris*, an endotoxin-free host system for recombinant protein production, has been used to produce scFv-h3D6, both in flask and in a fed-batch bioreactor. Comparison of the thermal stability of the obtained protein with that from *E*. *coli* showed no differences. Opposite to the case of the protein obtained from *E*. *coli*, no disulfide scrambled conformations or LPS traces were detected in that produced in *P*. *pastoris*. Cytotoxicity assays in SH-SY5Y neuroblastoma cell-cultures demonstrated that proteins from both expression systems were similarly efficient in precluding Aβ-induced toxicity. Finally, the 3xTg-AD mouse model was used to test the therapeutic effect of both proteins. Quantification of Aβ levels from cortex and hippocampus protein extracts by ELISA, and Aβ-immunohistochemistry, showed that both proteins reduced Aβ burden. This work demonstrates that scFv-h3D6 obtained from *P*. *pastoris* shows the same benefits as those already known for that obtained from *E*. *coli*, with multiple advantages in terms of recombinant production and safety.

## Introduction

Immunotherapy has recently emerged as a promising approach to treat numerous diseases including cancer, autoimmune disorders, transplant rejection and cardiovascular diseases [1]. The accumulation of the Amyloid-β (Aβ) peptide is the seed that initiates the disease process in Alzheimer’s disease (AD) [2, 3]. The pathogenicity of the oligomeric forms of the Aβ peptide [4, 5] led to the idea of designing new therapies to reduce Aβ burden. In that sense, antibodies are of great interest as they are an excellent paradigm for the design of high-affinity, protein-based binding reagents [6].

Specific monoclonal antibodies (mAbs) raised against the N-terminal region of Aβ were first described by Solomon and Frenkel [7–10]. In the early 2000s, passive immunization with antibodies against Aβ quickly showed promising results: reduction of amyloid deposition [11, 12], clearance of existing Aβ plaques, reduction of soluble peptide concentration [13] and reversion of Aβ-induced memory deficits [14–16]. However, when these antibodies advanced to clinical trials, their development had to be halted due adverse side-effects like vasogenic edema, meningoencephalitis or microcerebral hemorrhages, presumably induced by T-cell-mediated and/or Fc-mediated immune responses [17–19].

In 2002, Backsai *et al*. demonstrated that stereotaxic injection of a F(ab')2 fragment against Aβ led to the clearance of amyloid deposits in an AD mouse model, indicating that non-Fc mediated mechanisms were also involved in clearance [20]. Based upon these experimental and clinical observations, the use of humanized Aβ antibodies lacking Fc was proposed as a potential therapy for AD. Then, the redesign of antibodies by antibody phage display and their expression in bacterial systems played a key role in the generation and engineering of small antibodies [21–23]. Single chain variable fragments (scFv) are a recombinant format in which the V_H_ and V_L_ domains of immunoglobulins are joined with a flexible polypeptide linker preventing dissociation. They retain the specific, monovalent, antigen-binding affinity of the parent IgG, while showing improved pharmacokinetics for tissue penetration [24], and do not induce Fc-mediated activation of microglia.

In the recent years, scFv-h3D6, derived from the mAb bapineuzumab, has been shown to be a promising approach to treat AD. ScFv-h3D6 prevents Aβ-induced cytotoxicity by withdrawing Aβ oligomers from the amyloid pathway towards the worm-like (WL) one; a non-toxic pathway characterized by short and curved fibrils [25]. In addition, it has been proven to be effective in the triple transgenic 3xTg-AD mouse model of Alzheimer’s disease at the behavioral, cellular, and molecular levels. After a single intraperitoneal dose of scFv-h3D6, learning and memory deficits were ameliorated and a global decrease in Aβ oligomers was observed in the cortex and the olfactory bulb of young 3xTg-AD females. Indeed, scFv-h3D6 showed a great potential for treating other molecular features of AD, as the recovering of the non-pathological levels of apolipoproteins E and J [26, 27]. However, the expression of scFv-h3D6 in *Escherichia coli*, and especially its purification is an overwhelming process.

Although the scFv format is aglycosylated and so suitable for expression in *E*. *coli* [28], disulfide scrambling is favored in the reducing bacterial cytoplasm and proteins expressed in the insoluble fraction require a refolding process that can also generate disulfide scrambled conformations. In addition, proteins produced in *E*. *coli* are contaminated with endotoxins traces that, especially in the case of proteins with medical applications, must be removed. To overcome these hurdles, as well as to improve the yield, here, the yeast *Pichia pastoris*, an endotoxin-free host system for recombinant protein production, has been used to produce scFv-h3D6. As a eukaryote organism, *P*. *pastoris* has many of the advantages of higher eukaryotic expression systems such as protein processing, protein folding and posttranslational modification, while being as easy to manipulate as *E*. *coli*. It is also a faster, easier and less expensive system than mammalian cells [29].

Although several scFvs have been expressed in *P*. *pastoris* (i.e.[30–32]), here, the production of an anti-Aβ antibody fragment is shown for the first time. Two variants with different N-terminal sequence were generated in *P*. *pastoris* and, after determining the homogeneity depending on the protease cleavage performed during expression, the best one was selected and purified. In addition, the feasibility of translation to production for manufacturing purposes in a bioreactor was demonstrated. Comparison of the thermal stability of the obtained protein with that from *E*. *coli* showed no differences. Opposite to the case of the protein obtained from *E*. *coli*, that produced in *P*. *pastoris* showed no disulfide scrambled conformations or LPS traces, and remained aglycosylated. Cytotoxicity assays in SH-SY5Y neuroblastoma cell-cultures demonstrated that both proteins were similarly efficient in precluding Aβ-induced toxicity. Finally, the 3xTg-AD mouse model was used to assess the therapeutic effect of both treatments. Quantification of Aβ levels from cortex and hippocampus protein extracts by ELISA and Aβ-immunohistochemistry showed that both proteins reduced Aβ burden. Therefore, the protein obtained from *P*. *pastoris* is efficient and safe.

## Materials and methods

### Cloning

*ScFv-h3D6* gene was inserted in the pPicZαA vector (Invitrogen) in restriction sites *EcoRI* and *NotI* (New England Biolabs). To do so, an *EcoRI* target site had to be generated by PCR upstream of the *scFv-h3D6* gene (Oligonucleotides were purchased at Invitrogen). Single nucleotide mutations were performed using QuickChange Lightning Site-Directed Mutagenesis kit (Agilent Technologies). Ligation and PCR products were transformed into XL1Blue *E*. *coli* strain and grown on low-salt LB-Zeocine (Ibian Technologies) (vector resistance) agar plates. After extraction and purification of the plasmid, it was linearized by *PmeI* (New England Biolabs) restriction before transformation into *P*. *pastoris*.

### *P*. *pastoris* transformation and selection

The linearized DNA was transformed into competent *P*. *pastoris* KM71H cells by electroporation using *Electro Cell Manipulator Precision Plus* (BTX ECM 630). Transformant cells were grown on YPDS-Zeocine agar plates and screened for their ability to grow in increasing concentrations of Zeocine up to 10 mg/mL.

### Protein expression in *P*. *pastoris*

For protein expression tests, transformed *P*. *pastoris* cells with high resistance to Zeocine were grown in shake flasks containing 100 mL of buffered glycerol complex medium (BMGY, 1% yeast extract, 2% peptone, 100 mM potassium phosphate buffer at pH 6.0, 13.4 g/L YNB, 4x10^-4^ g/L biotin, 10 g/L glycerol and 100 μg/mL Zeocine) at 30°C and 250 rpm until an OD_600_ of 2–6 was reached. Then, the cell culture was centrifuged (3,000xg, 5 min, room temperature (RT)) and resuspended in 20 mL of BMMY (methanol instead of glycerol in BMGY). The medium was supplemented with methanol at a final concentration of 0.5% (v/v) every 24h. Expression was followed for five days. In the case of larger volumes of expression, 10 mL of BMGY were inoculated with transformed KM71H cells. After 16-18h of growing at 30°C and 250 rpm, these 10 mL were transferred to 1L of BMGY. When the OD_600_ reached 2–6, the cell culture was centrifuged (3,000xg, 5 min, RT) and resuspended in 200 mL of BMMY. Methanol was supplemented every 24h and expression was carried out for 48h.

### Large-scale production in *P*. *pastoris*

Fermentation was carried out at Bioingenium SL facilities. Fresh colonies were picked from YPD-Zeocine plates and grown overnight in 300 mL of BMGY medium. A 100-mL culture was transferred to a bioreactor (Applikon biobundle 5L, Applikon biotechnology), with 3L of fermentation basal salts medium supplemented with PTM_1_ Trace Salts. The bioreactor conditions were optimized by modification of [33]. The temperature and pH were set at 30°C and 6, respectively. Dissolved oxygen levels were fixed to a setpoint of 25% of saturation by regulating agitation at approximately 800 rpm. After complete consumption of glycerol in the medium (24h), a methanol fed-batch phase was initiated by adding methanol to reach a maximum concentration of 1% (v/v) (7.9 g/L). After 48 of induction (72h of total production), the culture was harvested and supernatant was kept for protein purification.

### ScFv-h3D6-Pp purification

Protein was secreted into the medium. After 48h of expression, cell culture was centrifuged (3,000xg, 10 min, RT). Supernatant was kept, the pH adjusted to 7.4 to facilitate protein precipitation (pI = 7.9), and ammonium sulfate was slowly added in agitation up to 50% (w/v). After 14-16h (o/n) at 4°C, the sample was centrifuged at 100,000xg for 1h at 4°C (Optimal LT X-100 Ultracentrifuge. Beckman Coultier). The pellet was resuspended in 10mM Na_2_HPO_4_, pH 6.5, and dialyzed for 24h (4 X 5L buffer changes). Then, a cationic exchange chromatography with a linear gradient of increasing NaCl concentration was performed. Finally, the protein was dialyzed to PBS, pH 7.4, and stored at -20°C until its use.

### Proteomics

Mass spectrometry (MS) analyses were carried out in the Proteomics facility at the UAB using a MALDI-TOF UltrafleXtreme (Bruker Daltonics). Sample preparation for each test was performed as follows:

**Peptide Mass Fingerprinting**: SDS-PAGE bands were cut and unstained with 50 mM ammonium bicarbonate/50% ACN. For disulfide bond reduction, samples were incubated with 10 mM DTT for 30 min at RT. For aquilation, samples were incubated with 25 mM iodoacetamide for 30 min at RT in the dark. Then, samples were digested with trypsin: 50 ng/sample (sequencing grade-Promega), 4h at 37°C. Finally, samples were eluted with H_2_O/50% acetonitril/0.2% TFA.

**Detection of disulfide bridges**: 30 μL of protein samples at 1.5 μg/μL were partially denatured with urea: 30 μL of sample +15 μL 4M urea, 2h at 30°C. Then, the samples were digested with 800 ng of trypsin, dissolved in 25 μL of 0.1M ammonium bicarbonate, for 4h at 30°C, and matrix-assisted laser desorption/ionization time-of-flight (MALDI-TOF) analysis was performed. Then, 2-μL samples were reduced with 0.05M DTT for 1h at RT and MALDI-TOF analysis was performed again.

**Mass spectrometry**: Protein samples were dialyzed by drop dialysis: 2-μL samples were dialyzed against 20 mL of 50 mM (NH_4_)HCO_3_ for 30 min at RT using a 0.025 μm pore membrane (Millipore). Then, samples were diluted 1/5 with milliQ-H_2_O, mixed 1:1 sample:matrix (2,6-dihidroxiacetophenone acid), and 1 μL deposited onto a ground steel plate. Analyses were performed using a linear method and an accelerating voltage of 25kv.

For peptide mass fingerprint (PMF) analyses samples were directly mixed 1:1 sample:matrix (α-cyano-4-hydroxycinnamic acid) and 1 μL of sample was deposited onto a ground steel plate. Samples were analyzed using a reflectron method with an accelerating voltage of 25kv. All the MALDI-TOF analyses were calibrated using external references (Bruker Daltonics).

### Lipopolysaccharides detection

Endotoxin units (EU) concentration (EU/mL) of the purified protein was determined by Pierce LAL Chromogenic Endotoxin Quantitation Kit (Thermo Scientific), following manufacturer’s instructions.

### ScFv-h3D6-Ec expression and purification in *E*. *coli*

Protein expression was carried out using pET28a (+) vector and *E*. *coli* BL21 strain. Induction with 0.5 mM IPTG (isopropyl *β*-D-thiogalactopyranoside) was performed at OD_600_ = 0.7 and incubation in the shaker at 20°C for 18h. After three freeze—thaw cycles, the cellular pellet was sonicated for 5 min, at 70% duty cycle and output 9 (Sonifier 450, Branson). The protein was obtained by solubilizing the insoluble fraction in denaturing buffer (100 mM Tris-HCl, 10 mM GSH, pH 8.5, and 8M urea) and refolding by dilution (1:10) in ice-cold refolding buffer (100 mM Tris/HCl, 100 mM L-arginine and 0.15 mM GSSG, pH 8.5) for 48h. Then a cationic exchange chromatography (Resource S6, GE Healthcare) using 5 mM Na_2_HPO_4_ pH 6.5 buffer and a gradient up to 15% of 5 mM Na_2_HPO_4_, 1M NaCl, pH 6.5 was performed. This chromatography was used to completely purify the protein and also to fractionate the native state and the disulfide scrambled forms. Finally, because proteins purified from *E*. *coli* contain lipopolysaccharides that are toxic to cell cultures, these were removed from the protein by using Detoxi-Gel Endotoxin Removing columns (Thermo Scientific). The buffer was changed to PBS using PD-10 Desalting Columns (GE).

### Secondary structure determination by Circular Dichroism (CD)

Protein secondary structure was monitored at different temperatures by far-UV CD spectroscopy from 260 nm to 190 nm in a Jasco J-715 spectrophotopolarimeter. Protein concentration was 20 μM, and 20 scans were recorded at 50 nm min^-1^ (response 2s) in a 0.2 cm pathlength cuvette.

### Thermal denaturation

Thermal denaturation was followed up by far-UV CD spectroscopy at 218 nm (Jasco J-715) and tryptophan fluorescence emission at 338 nm (Cary Eclipse, Varian), both at 20 μM protein concentration and 1°C min^-1^ heating rate.

### Transmission electron microscopy (TEM)

To visualize the aggregation extent and morphology of the scFv-h3D6-Ec and scFv-h3D6-Pp aggregates, incubation of 100-μM samples was carried out at 37°C for 48h. Then, samples were 1:10 diluted in PBS and quickly adsorbed onto glow-discharge carbon-coated grids. TEM was performed in a Jeol 120-kV JEM-1400 microscope, using 1% uranyl acetate for negative staining.

### Aβ preparations

Aβ_1–42_ synthetic lyophilized peptide (Bachem), was dissolved at 1 mM in HFIP (1,1,1,3,3,3-hexafluoro-2-isopropanol) (Sigma-Aldrich). Then, aliquots of 30 μL were prepared and HFIP was removed by vacuum drying in a SpeedVac (Savant instruments), and stored at −20°C. For TEM analysis, each aliquot was resuspended with 6 μL of DMSO (Sigma-Aldrich) and subsequently diluted to 300 μL (100 μM) with PBS and co-incubated with scFv-h3D6 variants at 37°C for 48h. For cell-culture cytotoxicity assays, phenol-red free DMEM (Gibco) was used instead of PBS and resuspended Aβ was incubated for 24h at 4°C before the addition to the wells [34], and then incubated at 37°C for 48h in the cell culture alone or together with the scFv-h3D6 variants.

### Cell culture and viability assays

The SH-SY5Y human neuroblastoma cell-line (RRID: CVCL_0019) was grown in serum-supplemented medium in 5% CO_2_ at 37°C. DMEM/F-12 (1:1) +GlutaMAX^™^ (Gibco) was supplemented with 10% fetal bovine serum (Sigma), 1% MEM non-essential amino acids (Gibco) and 1% mix of antibiotics: penicillin, streptomycin, and anti-fungal amphotericin (Gibco). 10,000 cells/well were plated in 96-well plates (Life Technologies) and incubated for 24h to allow cell attachment. Then, medium was changed and cells were treated with Aβ oligomers (10 μM) and/or scFv-h3D6 variants (0, 2.5, 5, 7.5 and 10 μM). Because of the method of preparation of the Aβ peptide, 2% (v/v) DMSO remained in the initial solution (0.2% in the well) and all samples, including controls, contained the same percentage of DMSO during incubation, as well as the same medium and buffer composition. After 48h of incubation, viability assay EZ4U (Biomedica) was performed following the manufacturer’s instructions. Each condition consisted of three replicas per experiment, and four independent experiments were performed. Data are presented as the percentage of viability for each condition compared to the untreated cells.

### Mice treatment

All the experiments were approved by the UAB Animal Research Committee and the Government of Catalonia, and performed in accordance with the Guide for the Care and Use of Laboratory Animals published by the US National Institutes of Health. Five-month-old triple-transgenic (3xTg-AD) mice females harboring *PS1/M146V*, *APPSwe* and *tauP301L* transgenes and non-transgenic (NTg) mice with the same genetic background (B6129SF2/J) (both purchased from the Jackson Laboratories and stablished at the UAB animal facility) were used. Animals of the same genotype and sex were maintained in cages (Makrolon, 35 × 35 × 25 cm) under standard laboratory conditions (food and water *ad lib*, 22 ± 2°C, 12h light:dark cycle starting at 08:00). Animals (n = 6 each group) received a single intraperitoneal dose of 100 μg of scFv-h3D6-Ec, scFv-h3D6-Pp or vehicle (PBS). Five days after administration, animals were anesthetized with inhaled isofluorane (1% in O_2_), sacrificed, and brains were collected and dissected. One hemisphere was kept for histological analysis and the other was used for protein extraction.

### Protein extracts

Protein extraction was performed by centrifugation of brain subregions homogenates. Briefly, frozen tissues of cortex and hippocampus from 5-mo-old 3xTg-AD and NTg mice were weighted and mechanically homogenized in ice-cold TBS-1% Triton X-100 solution supplemented with protease inhibitors (Roche tablets, reference 1836153) (8 μL solution/mg tissue). Then, samples were gently sonicated (1 cycle of 35 sec, at 35% duty cycle and output 4 in a Dynatech Sonic Dismembrator ARTEK 300 with the smallest tip) and centrifuged at 100,000g for 1h at 4°C. Supernatants were aliquoted and stored at −80°C until its use.

### Aβ_42_ ELISA

Brain extracts were used to analyze differences in Aβ levels of each group by Aβ_42_ ELISA (Invitrogen). Procedure was performed according to the manufacturer’s protocol. Data obtained were normalized by the total amount of protein in each extract measured by BCA assay (Pierce).

### Immunohistochemistry

Paraformaldehyde-fixed sections (cut at 10 μM) were deparaffined and treated with 70% formic acid for 20 min for epitope retrieval. To quench endogenous peroxidase, sections were incubated with 3% H_2_O_2_ in methanol for 10 min. Then, sections were washed and permeabilized in 0.1% Tween-PBS followed by blocking in 5% normal goat serum (Sigma-Aldrich), 5% BSA (Sigma-Aldrich) and 0.1% Tween-PBS. After blocking, sections were incubated with primary antibody 6E10 (ID: AB_564201) (Biolegend) overnight at 4ᵒC. The next day, sections were washed and immunostained with Mouse Extravidin Peroxidase Staining kit (Sigma-Aldrich) and developed by diaminobenzidine (DAB) substrate and stained with hematoxylin. Sections were cover-slipped using DPX mounting medium (Sigma-Aldrich) and examined in a Leica DMRB Microscope equipped with the Leica Application Suite (LAS) software.

### Statistics

Statistical analysis was performed using Graphpad 6 software. For cell viability assays, differences due to the treatment with scFv-h3D6-Ec or scFv-h3D6-Pp were assessed by two-way ANOVA test, analyzing changes due to protein concentration and protein variant. For comparisons of control groups (Aβ alone for cell viability assays or 3xTg-vehicle in the *in vivo* experiment) with treated conditions, unpaired t-test with Welch’s correction was performed. All data were expressed as means ± SEM values and a p value of <0.05 was considered to reflect statistical significance.

## Results and discussion

### Study of protein expression

Heterologous expression in *P*. *pastoris* can be either intracellular or secreted. Secretion requires the presence of a signal sequence on the expressed protein to target it to the secretory pathway. The vector used for the expression of scFv-h3D6 in *P*. *pastoris* was pPicZαA, which has a native *Saccharomyces cerevisiae* α-factor secretion signal (α-MF) that allows for efficient secretion of most proteins from *P*. *pastoris*. The processing of the α-MF signal sequence in pPicZαA occurs in two steps: the preliminary cleavage of the signal sequence by the *kex2* gene product and the subsequent shortening by the *STE13* gene product. Kex2 cleavage occurs between arginine and glutamic acid residues within the sequence Glu-Lys-Arg*-Glu-Ala-Glu-Ala, where * is the site of cleavage [35, 36]. The Glu-Ala repeats are further removed by the *STE13* gene product [37], but there are some cases where this cleavage is not efficient, and Glu-Ala repeats are left in the N-terminus of the expressed protein.

To insert the scFv-h3D6 gene in the pPicZαA vector, an *EcoRI* target was generated by PCR upstream of the *scFv-h3D6* gene. Then, the *scFv-h3D6* gene was introduced in the pPicZαA vector through *EcoRI* and *NotI* restriction sites. As a consequence of this procedure, a Phe residue was generated in the protein sequence (…EKREAEAE**F**EVQL…). Due to the bulky dimensions and hydrophobicity of this residue, and the difficulties these features could generate, mainly in terms of folding and immunogenicity, sequence modification was performed. Two variants were constructed: EAEA (…EKREA**EA**QL…) and EAEV (…EKREA**EV**QL…). In both variants, the EFEV sequence, which is not necessary for the protease processing, was removed, leading to a much closer location of the scFv-h3D6 N-terminus to the protease target site.

Both constructs were transformed into the Mut^S^ strain KM71H and zeocine selection was performed. Protein expression tests were carried out and both proteins were efficiently secreted into the medium. From both variants (EAEA and EAEV) two forms of protein were detected depending on the efficiency of the STE13 cleavage (not shown).

**Fig 1:**
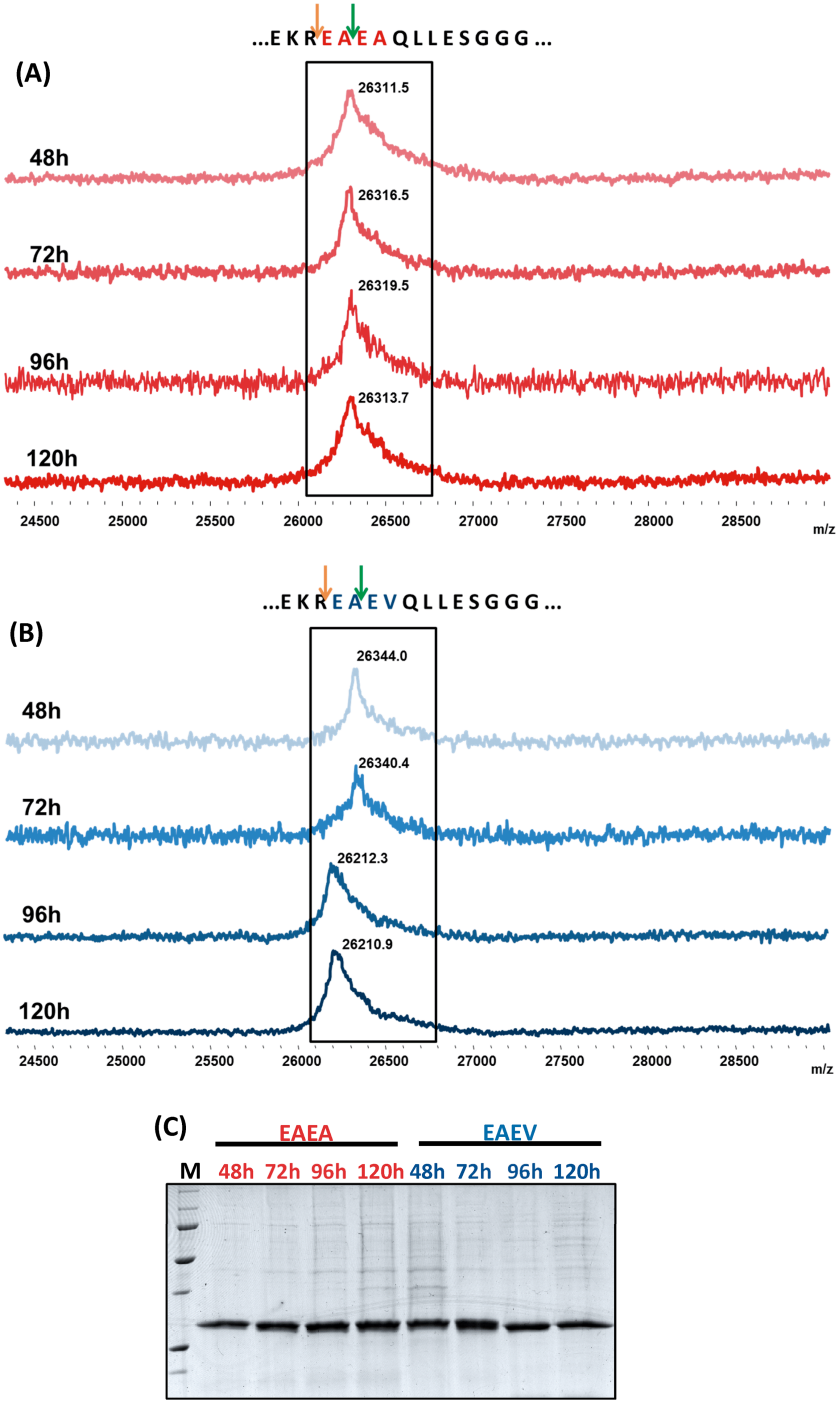
**Mass spectrometry MALDI-TOF analysis of protein expression at 48h, 72h, 96h and 120h after induction of EAEA (A) and EAEV (B) variants**. Orange arrows indicate the Kex2 cleavage site and green arrows indicate the STE13 one. While the molecular weight of the EAEV variant (26142.0 or 26342.2 Da depending on the protease cleavage site) changed during the induction period, the EAEA variant (26114.0 or 26314.2 Da) maintained a homogenous population. **(C) SDS-PAGE of the samples analyzed by mass spectrometry**. Secreted proteins rendered a good and similar expression yield.

To discern which of the two variants (EAEA or EAEV) was a better approach for obtaining a homogenous and pure scFv-h3D6, a longitudinal study of protein expression was performed for five days. Fig 1A and 1B show that while the molecular weight of the EAEV variant changed during the time-course of expression, as assessed by MALDI-TOF-MS, the molecular weight of the EAEA variant was always the same. This indicated that in the case of the EAEV variant, after the KEX2 processing, the STE13 cleavage was heterogeneously performed. On the other hand, the fact that the molecular weight of the EAEA variant was maintained indicated that the STE3 cleavage was either not performed or performed to an undetectable extent. Although both variants rendered a similar expression yield, as assessed by SDS-PAGE (Fig 1C), we decided to focus on the study of the EAEA variant to ensure homogeneity among batches.

### Protein purification from *P*. *pastoris*

When designing the protein constructs, no C-terminal *myc* epitope or polyhistidine tag for purification were added, as it is usually the case for proteins expressed in *P*. *pastoris*. Then, a stop codon was used just downstream the protein sequence to avoid the expression of C-terminal peptides included in the pPicZαA vector. As a therapy intended to treat AD patients, protein composition must be limited to the original sequence, without any other additional parts that could interfere with its the therapeutic effect or induce an immunologic response.

The major advantage of expressing heterologous proteins that are secreted into the medium is that *P*. *pastoris* secretes very low levels of its own proteins. Then, the secreted heterologous protein comprises the vast majority of the total protein in the medium [38], as was also the case for scFv-h3D6 (Fig 1C). The easiest way to purify the protein in this case was to centrifuge the cell culture and precipitate the protein in the supernatant with ammonium sulfate. Apart from a quick way to concentrate the protein, it is also a suitable manner to preserve it in case large volumes have been expressed and cannot be purified at once.

**Fig 2:**
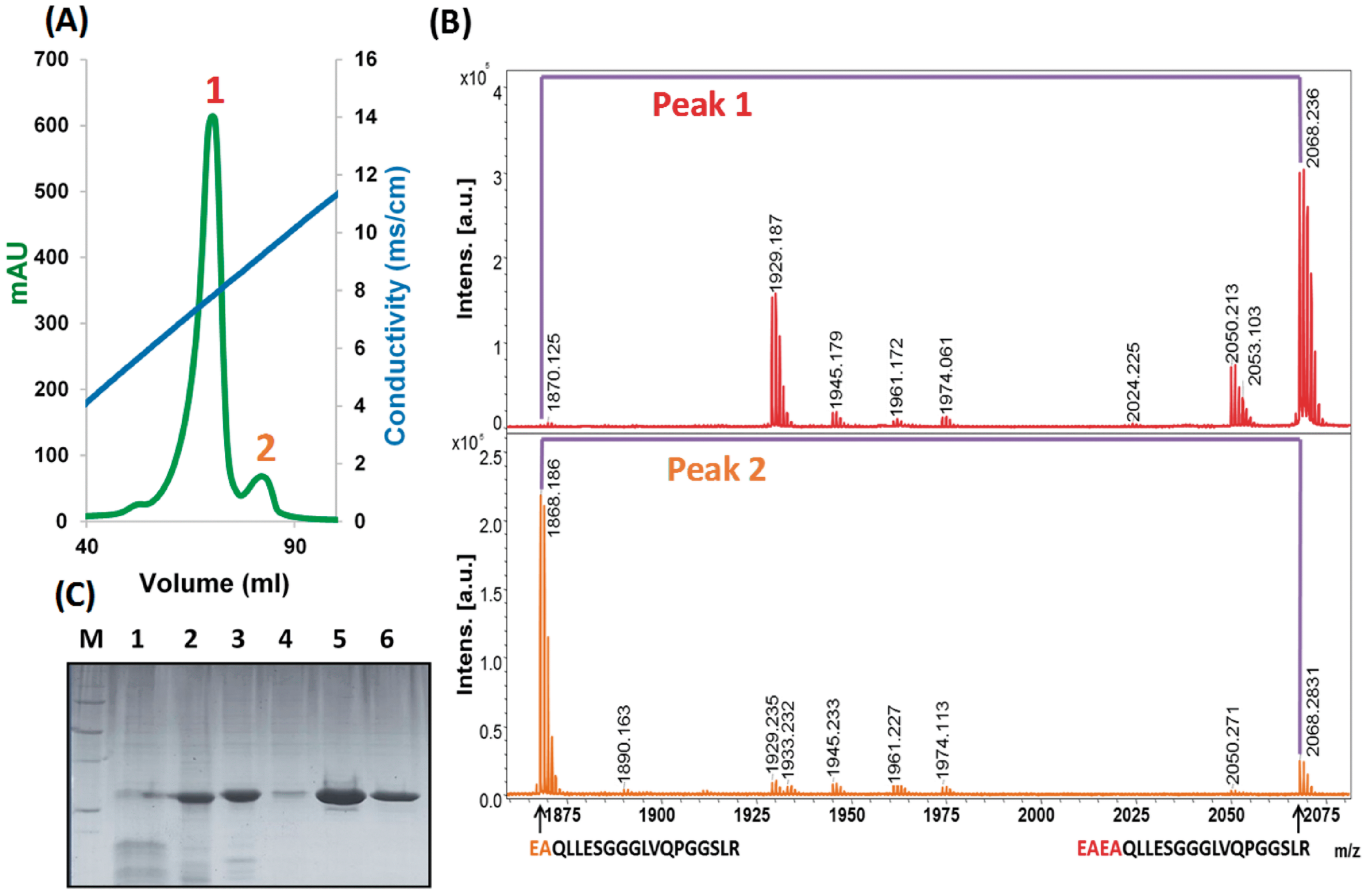
ScFv-h3D6-Pp purification. **(A) Cation Exchange Chromatography (CEX); (B) Peptide Mass Fingerprinting (PMF) analysis of each peak**. PMF analysis of the two differentiated peaks eluted after CEX indicated that the two fractions corresponded to the two possible protein variants depending on the STE13 protease cleavage site. **(C) SDS-PAGE of scFv-h3D6-Pp purification**. (M) Molecular weight marker; (1) Supernatant after ammonium sulfate precipitation; (2) Pellet after ammonium sulfate precipitation; (3) Sample before Cationic Exchange chromatography (CEX); (4) CEX flow through; (5) CEX elution of the mean peak; (6) PBS-dialyzed scFv-h3D6-Pp.

After centrifugation, pellet resuspension, and dialysis, a single cationic exchange chromatography was performed. As can be observed in Fig 2A, two differentiated peaks were eluted. PMF analysis indicated that the two fractions corresponded to the two possible variants depending on the STE13 protease cleavage site (Fig 2B), with the first eluted and most abundant peak corresponding to the EAEA variant and the second one to the EA one. Therefore, the small proportion of protein that was cleaved by STE13 could be fractioned from the most abundant EAEA form. This makes possible to easily achieve the isolation of a pure and conformationally homogenous protein. Then, as it constituted the majority of the production, we decided to focus our study on the EAEA variant, from now on called scFv-h3D6-Pp.

### ScFv-h3D6-Pp large scale production in *P*. *Pastoris*

**Fig 3:**
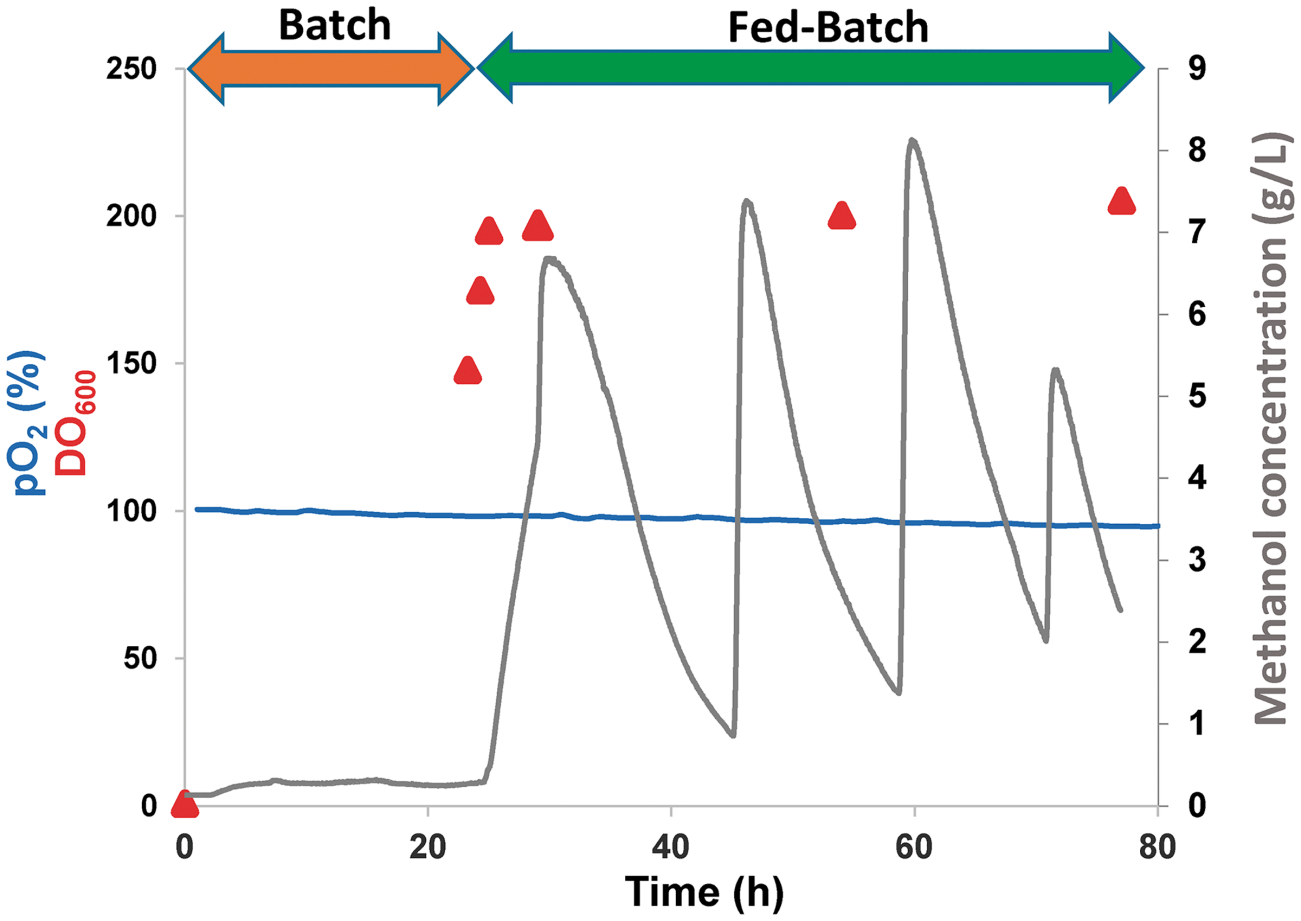
Large-scale fermentation profile. Oxygen concentration (expressed as the percentage of air saturation), cell grow (OD_600_) and methanol concentration (mg/mL of culture) during batch and feed-batch phases. Culture was induced 24h after the batch phase started. During the fed-batch phase, methanol was added periodically to reach a maximum concentration of 1% (v/v) (7.9 g/L).

To demonstrate that the production of scFv-h3D6-Pp could be easily scaled-up, so that translation to production for manufacturing purposes would become achievable, fermentation was performed in a 5L-bioreactor and then the protein was purified. Culture conditions were maintained as described in the Materials and methods section. Fermentation was performed in two phases. First, a batch phase with glycerol in the medium allowed for an exponential cell growth, and after 24h (OD_600_ = 148), when glycerol was almost exhausted, a 48h fed-batch phase started with the injection of methanol for inducing protein expression (Fig 3). Methanol was injected periodically depending on its concentration in the medium to avoid cell toxicity. After 48h of induction (72h of total production), the culture was harvested and the protein in the supernatant precipitated with ammonium sulfate. After purification by CEX chromatography, the final purification yield was 20.5 mg per initial L of growth culture. In *E*.*coli* production, the purification yield was 2.9 mg per L (Table 1), so we have considerably increased the production yield. Table 1 shows that in the purification from *E*. *coli*, most of the protein is lost during the refolding step; whereas in the case of the purification from P. pastoris, this occurs in the precipitation step despite it was optimized by adjusting the pH of the medium (5.4) to a value (7.4) close to the pI of the protein (7.9), prior to the addition of the ammonium sulfate.

**Table 1:** Comparison of the purification yields of scFv-h3D6-Ec and scFv-h3D6-Pp. Values were obtained from the purification of 1L of initial growth culture. Values are presented as mean±SD.

scFv-h3D6-Ec		scFv-h3D6-Pp	
Purification step	Total protein (mg)	Purification step	Total protein (mg)
Insoluble fraction	119.9±0.2	Medium (48h expression)	337.8±9.2
		AS Supernatant	120.0±2.5
After refolding/Before CEX	81.1±4.9	AS Pellet/Before CEX	206.4±18.8
Scrambled fraction	4.0±0.1		
Native protein	2.9±0.2	Pure protein	20.5±1.8

### Biophysical properties of scFv-h3D6-Pp compared to scFv-h3D6-Ec

**Fig 4:**
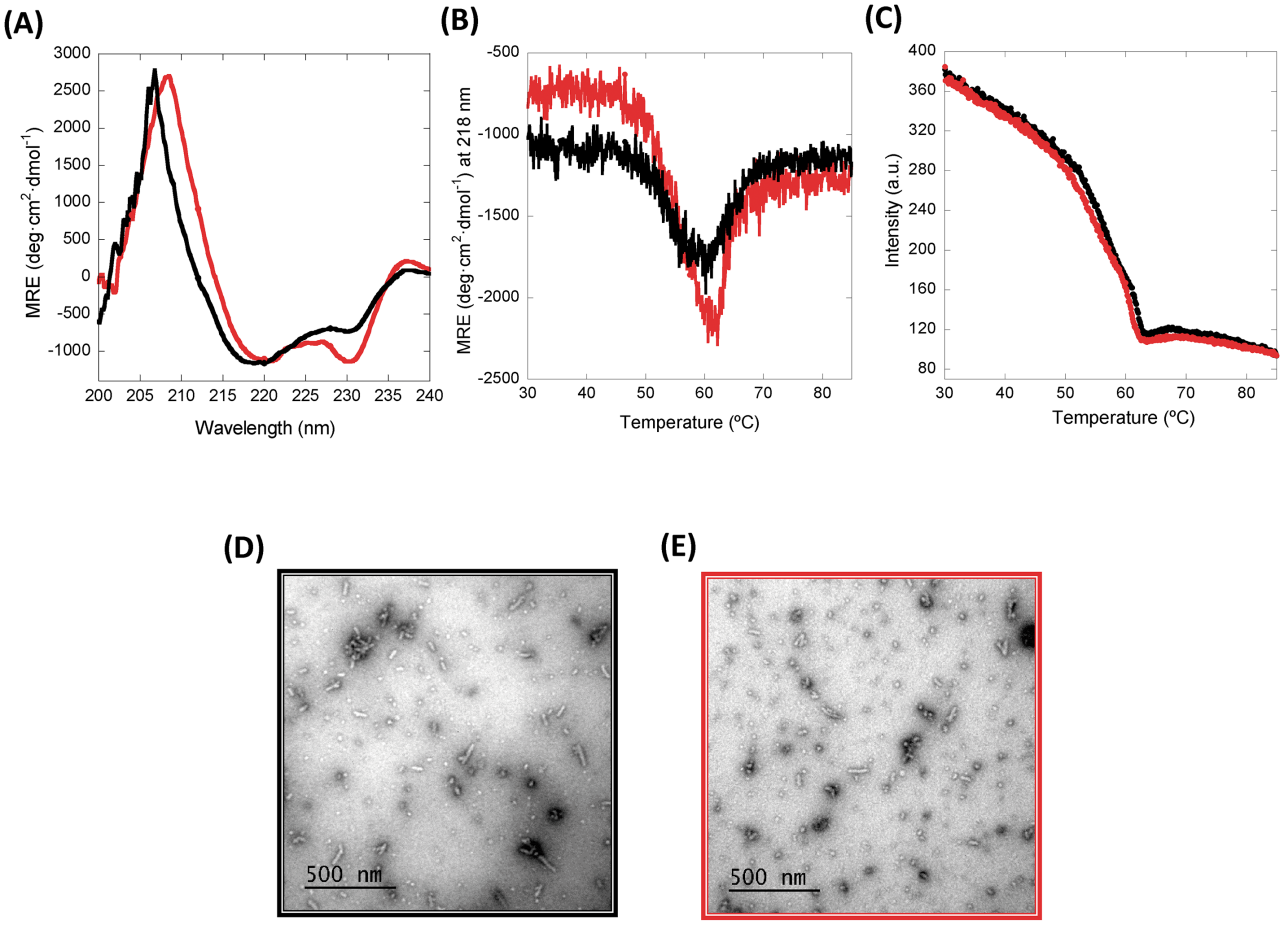
Protein characterization. **(A) Circular Dichroism (CD) spectra at 25°C; (B)Thermal denaturation followed by CD; (C) Thermal denaturation followed by Trp-fluorescence; (D) and (E) TEM micrographs**. Black: scFv-h3D6-Ec, Red: scFv-h3D6-Pp. CD analysis showed that the β-conformation characteristic of the immunoglobulin fold is maintained, albeit some differences in the interferences due to the Trp residues in the core of each domain are somehow higher in the scFv-h3D6-Pp spectrum. However, no differences in terms of thermal stability were observed and, therefore, it can be assumed that both molecules are equally folded. As expected, worm-like fibrils, behind the protective effect of scFv-h3D6, are formed upon thermal denaturation in both cases, so that the therapeutic effect should remain.

To characterize the novel protein purified from *P*. *pastoris*, scFv-h3D6-Ec was used for comparative purposes. It is described that the CD spectra of the scFv-h3D6-Ec shows a peculiar minimum at 230 nm and a positive shoulder at 237 nm, corresponding to the interference of the Trp residue in the core of the V_L_ and V_H_ domain, respectively [25, 39]; apart from the characteristic minimum at 218 nm and maximum at around 200 nm for a β-fold (Fig 4A). These interferences are more intense for scFv-h3D6-Pp; however, it is rather difficult to find the rationale behind the effect in the far-UV region (260–190 nm) of an aromatic residue that typically renders ellipticity in the near-UV region (at around 290 nm). So, our hypothesis is just that differences between the N-terminus of both proteins, which contain the sequence MEVQLL for the *E*. *coli* variant and EAEAQLL for the *P*. *pastoris* one, could somehow affect the packing of the molecule, accounting for such an effect. In any case, the β-conformation characteristic of the immunoglobulin fold is kept.

To check whether these differences might affect conformation, thermal denaturation was followed by CD and Trp-fluorescence spectroscopies (Fig 4B and 4C). The profiles were the same in both cases, indicating that the protein obtained from *P*. *pastoris* shows no differences with that from *E*. *coli* in terms of thermal stability and, therefore, we can assume that both molecules are equally folded. As expected, worm-like fibrils, behind the protective effect of scFv-h3D6, are formed upon thermal denaturation in both cases (Fig 4D and 4E), so the therapeutic effect should remain.

### Advantages of scFv-h3D6-Pp as a therapeutic approach

**Fig 5:**
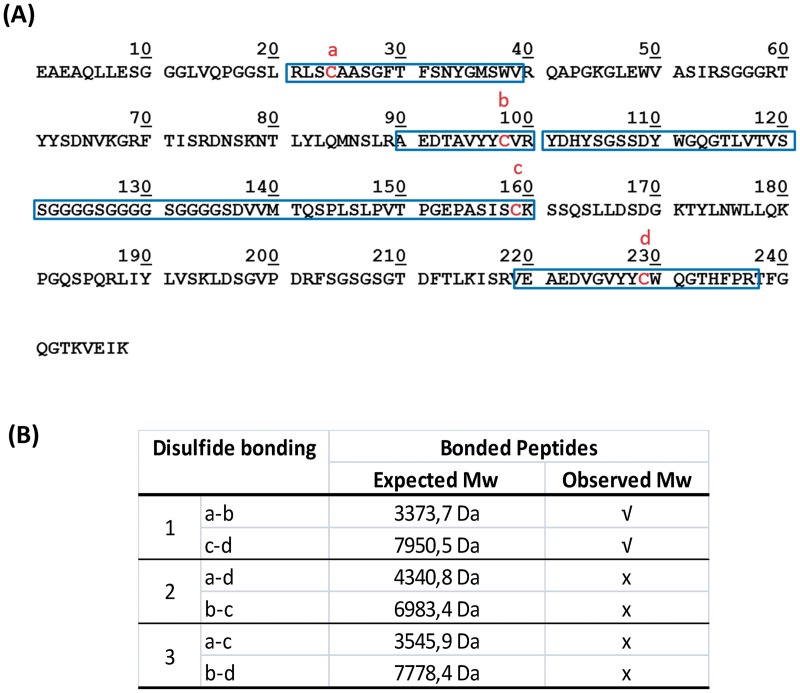
**(A) Sequence of scFv-h3D6-Pp protein**. Cysteine residues are indicated in bold red; tryptic peptides containing cysteine residues are highlighted in a blue rectangle. **(B) Disulfide bonds present in the scFv-h3D6-Pp**. Expected Mw of the tryptic peptides involved in disulfide pairing for each possible disulfide bonding conformation. The peptides detected by MALDI-TOF MS are indicated.

One of the main drawbacks of *E*. *coli* as a host organism for protein expression and purification is due to protein expression in the insoluble fraction, as is the case for most scFvs in the literature [25, 40–43] and also for scFv-h3D6-Ec. In this case, the insoluble fraction needs to be chemically solubilized and proteins, which are denatured, must be refolded. In this process, some disulfide scrambled conformations can occur. Separating scrambled conformations can become an arduous process, especially due to changes in conformation and the subsequent decrease in the purification yield. In addition, some traces of scrambled conformations can remain in the sample or, even after a good fractionation of the native state, appear as a consequence of the reshuffling of the disulfide bonds. These scrambled conformations, even in a very-low concentration, are prone to aggregation and consequently are thermodynamically trapped so that the equilibrium shifts from the native state to the scrambled forms. *P*. *pastoris* has many of the advantages of higher eukaryotic expression systems such as protein processing and protein folding. In this case, scFv-h3D6-Pp was secreted into the medium as a soluble form. In order to ensure that this soluble form corresponded to the native conformation, we checked the configuration of its disulfide bridges by analyzing the tryptic digestion of the non-reduced protein by MALDI-TOF-MS. Only the peptides corresponding to the native disulfide bonding were detected (Fig 5), indicating that after purification, scfv-h3D6-Pp was perfectly folded, and no scrambled conformations traces were present.

**Fig 6:**
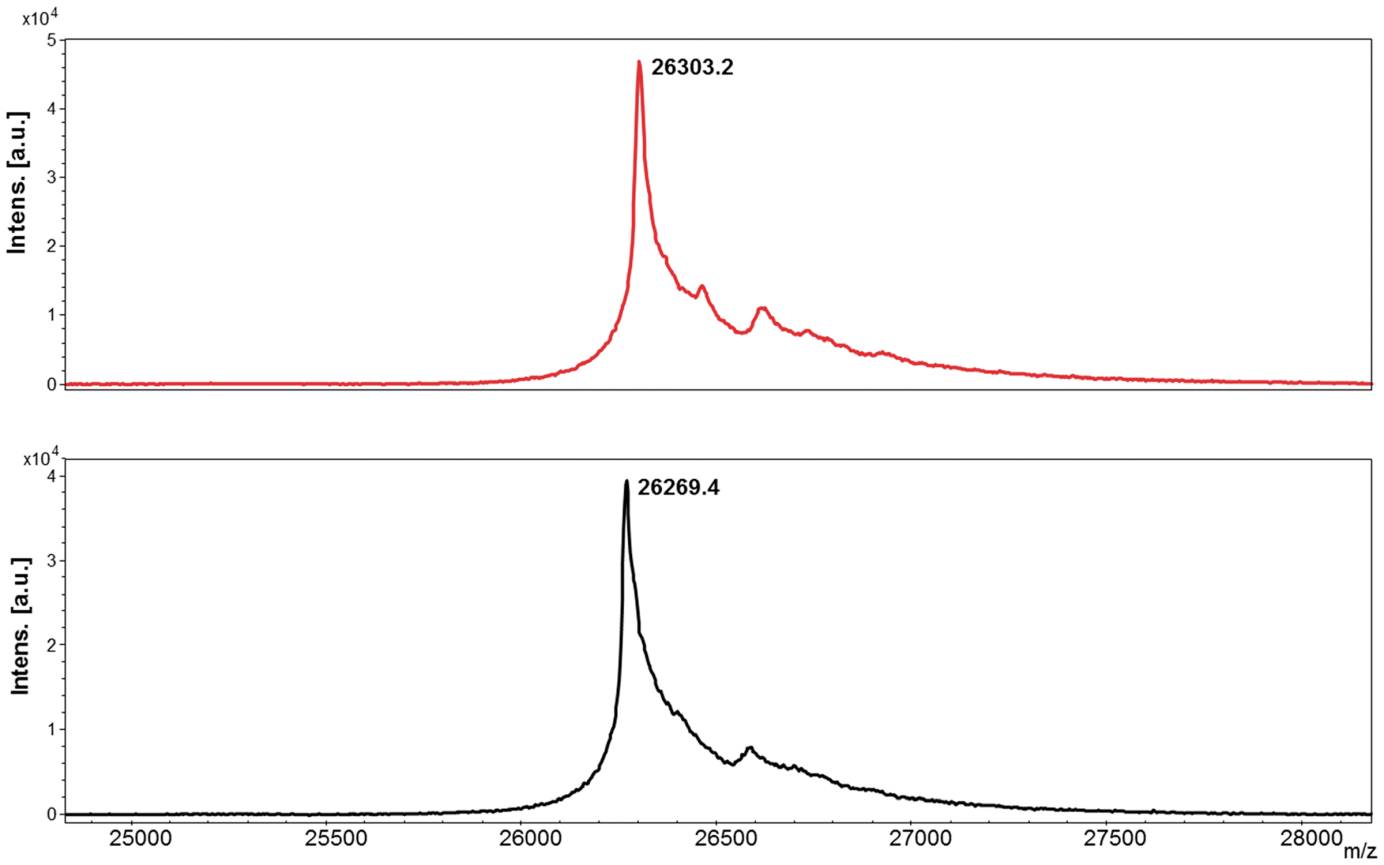
Mass spectrometry analysis of scFv-h3D6-Pp (red) and scFv-h3D6-Ec (black). Molecular weight analysis determined that no N-glycosylation either O-glycosylation was added to the protein as a posttranslational modification, as the molecular weight corresponded to the amino acid sequence alone (scFv-h3D6-Pp: 26314.2 Da and scFv-h3D6-Ec: 26273.2 Da).

On the other hand, one of the main drawbacks of expressing heterologous proteins in eukaryotic systems can be the posttranslational modifications carried out by the host organism, especially glycosylation. Contrary to *Saccharomyces cerevisiae*, *Pichia* may not hyperglycosylate secreted proteins. In the case of N-linked glycosylation, both *Saccharomyces* and *Pichia* have a majority of the N-linked glycosylation as high-mannose type; however the length of the oligosaccharide chains post-translationally added to proteins in *Pichia* is much shorter than those in *Saccharomyces* [44]. On the other hand, very little O-glycosylation has been observed in *Pichia* [45]. Predictor NetNGlyc 1.0 Server showed no targets for N-glycosylation within the sequence of scFv-h3D6-Pp, but predicted one signal sequence for O-glycosylation. Molecular weight analysis by MS (Fig 6) determined that no glycosylation was added to the protein as a posttranslational modification, as the molecular weight corresponded to the amino acid sequence alone.

Finally, using Gram-negative bacteria, such as *E*. *coli*, to obtain recombinant proteins with biomedical applications presents a great obstacle: the presence of endotoxins. Although systems for removal of lipopolysaccharide (LPS) are implemented [46–48], they deeply decrease the purification yield and increase the purification costs. Some *E*. *coli* strains have recently been engineered to produce modified versions of LPS that are supposed to avoid the endotoxin response [49]. *ClearColi* cells contain lipid IVA instead of LPS. Lipid IVA is incapable of inducing an endotoxic response in human immune cells, but may act as an endotoxic activator in other mammalian hosts such as mouse. However, lipid IVA is much easier to remove from products than LPS, and so this strain facilitates the production of therapeutic proteins in *E*. *coli*. Here we have used the yeast *P*. *pastoris* that apart from being considered an endotoxin-free cell factory for recombinant protein production, as a eukaryotic system properly folds proteins (see above). To demonstrate that the scFv-h3D6-Pp is a secure medical device, the level of endotoxins in the purified protein was determined. According to FDA [50], the endotoxins limit for a medical device is dependent on the intended use of the device and what the device contacts (e.g., blood, the cardiovascular system, cerebrospinal fluid, intrathecal routes of administration, permanently implanted devices, and devices implanted subcutaneously). For medical devices, the limit is 0.5 EU/mL or 20 EU/device for products that directly or indirectly contact the cardiovascular and lymphatic systems. In the *in vivo* experiment (following section) the protein was administered intraperitoneally at a concentration of 0.5 mg/mL and a total volume of 0.2 mL (100 μg). The testing of remaining LPS traces was also performed at this concentration of protein. The concentration of endotoxins in the protein was EU/mL = 56.46±1.24, what supposes 11.29 EU in the administration, and consequently, is under the limit. Therefore, scFv-h3D6-Pp can be safely used for therapeutic purposes.

### Therapeutic effects of scFv-h3D6-Pp: Protective effects in cell culture and reduction of Aβ burden in the 3xTg-AD mouse-model

**Fig 7:**
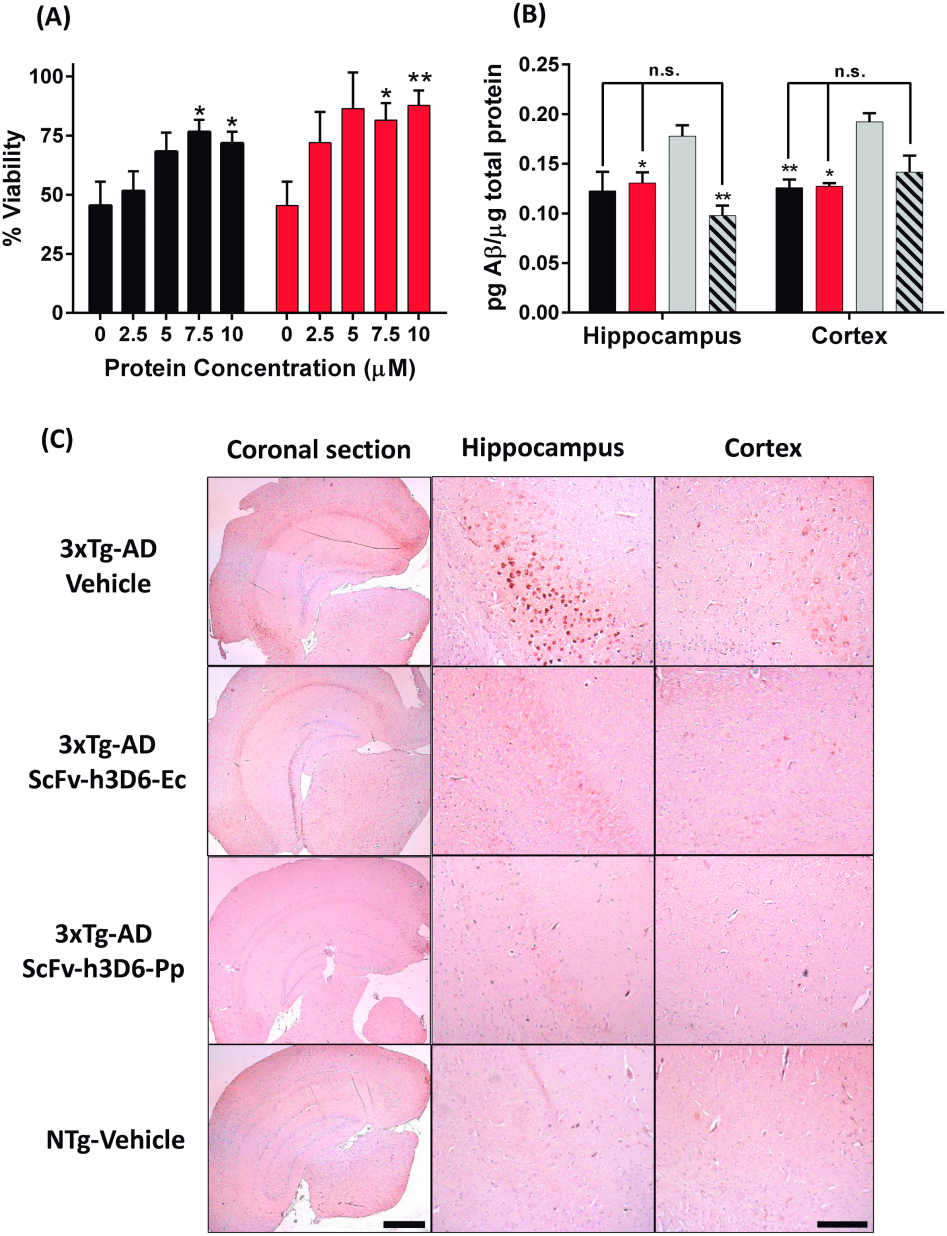
Therapeutic effects of scFv-hD6-Ec and scFv-h3D6-Pp. **(A) Viability assays in SH-SY5Y neuroblastoma cell line**. Cells were exposed to Aβ oligomers (10 μM) and different concentrations of scFv-h3D6-Ec or scFv-h3D6-Pp. Black: scFv-h3D6-Ec, Red: scFv-h3D6-Pp. Comparisons of each concentration of scFv-h3D6-Ec or scFv-h3D6-Pp with Aβ alone showed statistical significance for 7.5 and 10 μM in both cases, indicating efficiency of both treatments (unpaired t-test with Welch’s correction, * p<0.05. **p<0.01). **(B) Aβ**_**42**_ **ELISA of brain homogenates (Hippocampus and Cortex)**; Black: scFv-h3D6-Ec, Red: scFv-h3D6-Pp, Grey: 3xTg-AD-Vehicle, Stripped: NTg-vehicle. Both treatments recovered the non-pathological levels of Aβ. *, ** indicate significance compared to the 3xTg-AD-vehicle group (Unpaired t-test with Welch’s correction, *p<0.05, **p<0.01). Arrows indicate comparisons with NTg-vehicle group (n.s. indicates no significance). **(C) Aβ Immunohistochemistry of coronal sections**. Aβ-immunoreactivity decreased to similar levels as those in non-transgenic animals when 3xTg-AD were treated with scFv-h3D6-Ec or scFv-h3D6-Pp. Bar in panoramic coronal sections (2.5x zoom in) corresponds to 1 mm and in hippocampus and cortex sections (16x zoom in) to 200 μm.

Aβ oligomeric species are the crucial toxic species in AD [51, 52]. To test the therapeutic effects of the scFv-h3D6-Pp we studied its protective effect against Aβ oligomers’ neurotoxicity in the SH-SY5Y neuroblastoma cell-line. Aβ concentration was fixed to 10 μM (previously reported as toxic [25]) and protein concentration was tested in a range of concentrations (0, 2.5, 5, 7.5 and 10 μM). Fig 7A shows how both variants, from *E*. *coli* and from *P*. *pastoris*, are capable of blocking Aβ-induced toxicity in a concentration-dependent manner (two-way ANOVA p = 0.0016) with a marginal significance due to differences among mutants (two-way ANOVA p = 0.0508). On the other hand, when comparing each concentration of scFv-h3D6-Ec or scFv-h3D6-Pp with Aβ alone (unpaired t-test with Welch’s correction), statistical significance was reached in both cases for 7.5 and 10 μM concentrations (scFv-h3D6-Ec: for 7.5 μM p = 0.026 and for 10 μM p = 0.049; scFv-h3D6-Pp: for 7.5 μM p = 0.017 and for 10 μM p = 0.0067). These results indicated that both proteins are capable of recovering Aβ-induced toxicity, with scFv-h3D6-Pp showing a tendency to be more effective than scFv-h3D6-Ec.

Both proteins were also tested in the treatment of the 3xTg-AD mouse model. A single intraperitoneal dose of 100 μg of protein was administered to the following groups (n = 6): scFv-h3D6-Ec, scFv-h3D6-Pp and vehicle (PBS). A non-transgenic group (NTg) was also administered with vehicle in order to have a control of non-pathological levels of Aβ. After five days, animals were sacrificed. ELISA of brain extracts (Fig 7B) indicated that Aβ_42_ levels were decreased in cortex and hippocampus by both scFv-h3D6-Ec and scFv-h3D6-Pp. Statistical analysis of Aβ_42_ levels to determine differences was performed using unpaired t-test with Welch’s correction. Comparisons of the treated groups or NTg with 3xTg-AD-vehicle group showed p-values for cortex and hippocampus as follows: 3xTg-AD-scFv-h3D6-Ec (Cortex p = 0.011, Hippocampus p = 0.084), 3xTg-AD-scFv-h3D6-Pp (Cortex p = 0.005, Hippocampus p = 0.037), and NTg-vehicle (Cortex p = 0.075, Hippocampus p = 0.006). Therefore, scFv-h3D6-Ec was efficient in decreasing Aβ in the cortex and showed a tendency in the hippocampus, whereas scFv-h3D6-Pp was clearly efficient in both areas. It must be mentioned, however, that the Aβ levels in the cortex of the NTg-vehicle group did not reach significance when compared to the 3xTg-AD-vehicle ones, albeit the former levels were lower. On the other hand, no significance was reached when comparing 3xTg-AD-scFv-h3D6-Ec and 3xTg-AD-scFv-h3D6-Pp to NTg-vehicle, which indicates that treatments recovered the non-pathological Aβ_42_ levels in both areas.

Finally, immunohistological analysis of brain slices with the anti-Aβ antibody 6E10 (Fig 7C) also corroborated these results. Aβ-immunoreactivity decreased to similar levels as those in non-transgenic animals when 3xTg-AD were treated with scFv-h3D6-Ec or scFv-h3D6-Pp. Therefore, these results demonstrate that scFv-h3D6-Pp shows the same therapeutic benefits, or slightly better, as those already known for scFv-h3D6 obtained from *E*. *coli* [26, 27]. It is capable of withdrawing Aβ oligomers from the amyloid pathway and, this way, prevent their cytotoxicity. *In vivo*, scFv-h3D6-Pp significantly reduces Aβ burden, the increase of which is the most important hallmark of AD. This work validates scFv-h3D6-Pp as a therapy for AD with multiple advantages in terms of recombinant production and safety.

## Conclusions

ScFv-h3D6 has been demonstrated to be a promising approach to treat AD. Although its potential properties, scFv-h3D6 production in *E*. *coli* is limited by some bottlenecks like the presence of disulfide scrambled conformations generated in the refolding process and its contamination with endotoxins. In the present study, we present a simple and efficient system for production of scFv-h3D6 in *P*. *pastoris*.

One of the hurdles of expressing proteins in *P*. *pastoris* is the variability of protease action when they are secreted into the medium. Here we demonstrate that it is possible to obtain a pure and homogenous protein. Moreover, with an easy protein purification system, even though the protein sequence has no tags to facilitate purification. The scale-up process was also possible, what is important if this therapeutic approach is eventually used in the treatment of AD patients.

The obtained protein was demonstrated to be well-folded. In addition, LPS traces were under the limits set by FDA and no N- either O-glycosylation was performed by the posttranscriptional machinery of *P*. *pastoris*, assuring safety of the treatment in terms of avoiding an inflammatory response.

Finally, viability experiments with the neuroblastoma cell line SH-SY5Y demonstrated that the novel protein purified from *P*. *pastoris* is capable of avoiding Aβ-induced cytotoxicity as well as the protein from *E*. *coli*. *In vivo* experiments corroborated that there are no differences with the already known benefits of scFv-h3D6 obtained from *E*. *coli*. Therefore, *P*. *pastoris* constitutes an improved expression system to obtain scFv-h3D6 with therapeutic purposes. This new system must allow the limitations of scFv-h3D6 production not to interfere with its great potential as a therapy to treat AD.
